# Home mechanical ventilation and specialised health care in the community: Between a rock and a hard place

**DOI:** 10.1186/1472-6963-11-115

**Published:** 2011-05-23

**Authors:** Knut Dybwik, Erik W Nielsen, Berit S Brinchmann

**Affiliations:** 1 Department of Anesthesiology, Nordland Hospital, N-8092 Bodø, Norway; 2 Institute of Clinical Medicine, University of Tromsø, N-9037, Norway; 3 Faculty of Professional Studies, University of Nordland, N-8049, Norway

## Abstract

**Background:**

Home mechanical ventilation probably represents the most advanced and complicated type of medical treatment provisioned outside a hospital setting. The aim of this study was both to explore the challenges experienced by health care professionals in community health care services when caring for patients dependent on home mechanical ventilation, continual care and highly advanced technology, and their proposed solutions to these challenges.

**Methods:**

Using qualitative research methods, a grounded theory influenced approach was used to explore the respondents' experiences and proposed solutions. A total of 34 multidisciplinary respondents from five different communities in Norway were recruited for five focus groups.

**Results:**

The core category in our findings was what health care professionals in community health care services experience as "between a rock and a hard place," when working with hospitals, family members, and patients. We further identified four subcategories, "to be a guest in the patient's home," "to be accepted or not," "who decides," and "how much can we take." The main background for these challenges seems to stem from patients living and receiving care in their private homes, which often leads to conflicts with family members. These challenges can have a negative effect on both the community health caregivers' work environment and the community health service's provision of professional care.

**Conclusions:**

This study has identified that care of individuals with complex needs and dependent on home mechanical ventilation presents a wide range of immense challenges for community health care services. The results of this study point towards a need to define the roles of family caregivers and health care professionals and also to find solutions to improve their collaboration. The need to improve the work environment for caregivers directly involved in home-care also exists. The study also shows the need for more dialogue concerning eligibility requirements, rights, and limitations of patients in the provision and use of ventilatory support in private homes.

## Background

Home mechanical ventilation (HMV) probably represents the most advanced and complicated type of medical treatment provisioned outside a hospital setting [1,2]. This is especially apparent in the group of patients, consisting of both children and adults, who have tracheostomies and depend on specialised and costly care, monitoring, and ventilation support around the clock or for the majority of the day. In Norway, this group accounts for 7.8% of the total HMV patient population [3]. The remaining patients use non-invasive ventilation. The majority of HMV patients are given ventilation support in their homes but some live in public health care institutions or nursing homes. Family members are often involved in daily care and perform technical procedures [4]. These families are assisted by community health care services, especially when the patient is completely dependent on mechanical ventilation support or lives in a health care facility. A number of studies have investigated the strain put on family caregivers for this patient group, especially the pediatric patient population, but few studies have focused on identifying the challenges of caring for at-home HMV patients using the perspective of community health care services.

Generally, care for someone in his or her own home takes place in a different context from caring for him or her in a hospital, and requires a different approach [5]. Home care nurses use the terms "guest" or "professional" to characterise their relationships with patients and it seems impossible to be both at the same time [6]. Guests do not typically make demands [7] and the nurse must be aware of the patients' right to influence their own care, especially in their own home. Previous research shows that nurses believe their position of power and authority may be threatened or challenged when family members participate in specialised nursing care for family members dependent on highly advanced technology [8]. Health care professionals (HCP's) not recognising the parents of HMV technology-dependent children as experts may lead to conflicts [4]. Similar to family members, HCP's also experience fatigue, depression, and burnout, and very few professionals choose to work with this patient group [9]. High turnover rates among HCP's often lead to a dysfunctional relationship between family and health care providers [10], and in contrast to this, continuity in care is described as a success factor for a good and active life for the patient [11]. Community nurses can be dissatisfied with the hospitals' discharge planning because the nurses are given little opportunity for involvement and too little time for practical preparations [12,13]. Determining whether the discharging hospital or the receiving community has medical responsibility for at-home HMV patients may cause confusion [12]. In the UK, it has been speculated that community health care development has not kept up with the medical and technical advancements that make it possible to discharge children with complex needs from hospital [12].

The aim of this study was both to explore the challenges experienced by HCP's in community health care services when caring for HMV patients dependent on continual care and highly advanced technology, and their proposed solutions to these challenges. Several factors influenced our decision to conduct this study:

• Norway being no exception, little research has been conducted in this specific area.

• Specialised hospitals must recognise and be aware of these challenges to ensure a safe and successful transition from hospital to community care.

• Communities providing care for this patient group for the first time could benefit from learning about these challenges.

• This knowledge will be an important point of reference when health care authorities develop clinical guidelines for HMV, which is currently underway in Norway.

## Methods

### Study design

Qualitative research methods using a grounded theory influenced approach and focus groups were selected to explore the experience of the community health care services.

### Study setting

Organising and financing care of HMV patients varies between countries and health care systems. We studied the Norwegian public financial health system in which specialised hospitals establish ventilation support and follow-up even after hospital discharge. Community health care services, in cooperation with family caregivers, have responsibility for the daily care provided in the patient's home. In Norway, family members can be paid employees as part of the health care team caring for the HMV-dependent family member.

### Sampling

In classic grounded theory, the ongoing analysis steers the sampling and the data collection (theoretical sampling), but this can prove difficult to implement in practice. Strauss and Corbin state that, "as with all research, there is the ideal way of conducting a study and the practical way (or that for which one has to settle)" [14]. At first, we established contact with five communities from different regions in Norway that currently provide highly advanced care to HMV-dependent patients. We were ready to recruit more if necessary, but data collection ceased after 5 focus group sessions, when we determined that new information did not add new knowledge or insight (theoretical saturation).

### Recruitment

First, the home care administrators in each community were contacted via telephone to discuss their willingness to participate and an information letter was later sent. To extract a broad description of experience, home care administrators were asked to recruit all relevant HCP's involved in one way or another in care of tracheostomised and HMV-dependent patients; this being limited, however, to a maximum of 10 respondents in each group. Participation criteria included respondents with administrative or coordination responsibilities, and other personnel directly involved in practical care in the patient's residence. Unlicensed caregivers were also included. These caregivers had no formal health diploma, but instead received training and guidance from registered nurses, patients, and family members to perform HMV-related nursing tasks. Family members were not recruited as respondents. The characteristics of all 34 respondents are summarised in table 1.

**Table 1:** Characteristics of respondents, and the patients the communities cared for

Focus gr/community	Profession	Total patients*	Total years of experience with HMV	Patient's residence
1	RN/nurse manager, RN/previous nurse manager, RN, PA and 2 CNAs	1	9	1 Assisted-living facility

2	RN/nurse manager, RN/nurse manager, LVN, 2 CNA's and 4 RN's	3	39	2 Assisted-living facilities 1 Private residence

3	RN/Communal director, social educator/unit manager, critical care RN and 2 RN's	3	33	2 Assisted-living facilities 1 Private residence

4	RN/previous leader, social educator/unit manager, environmental therapist, child pedagogue, 2 PA's and 2 CNA's	5	48	5 Private residences

5	Child welfare social worker/unit manager, RN/case manager, RN/consultant, social educator and 2 RN's	2	20	2 Private residences

Abbreviation: RN: registered nurse, PA: personal assistant, CNA: certified Nursing Assistant, LVN: licenced vocational nurse

*all patients had tracheostomies and were ventilator dependent for either the entire or majority of the day

All of the five participating communities had extensive experience with this type of care. During the data collection period, a total of 14 HMV-dependent patients were cared for in the five communities. Two of the communities cared for a total of three HMV-dependent children, with the remaining 11 patients being adults (Table 1). All of the ventilator-dependent individuals had chronic neuromuscular disease (NMD), like, amyotrophic lateral sclerosis, Duchenne muscular dystrophy, infantile myofibromatose, limb-girdle muscular dystrophy, nemaline myopathy, spinal muscular atrophy type 1 and syringomyelia. Ages varied from eight to 78 and although the degree of disability varied, all were capable of being mobilised in a wheelchair during the day.

### Data collection

Focus groups have proven to be particularly useful for gaining thorough descriptions of knowledge, experience, priorities, and attitudes [15]. The spontaneity of group-based conversations may provide insight into topics that could be difficult to gain using other methods [16]. In this study, the number of respondents in each of the five focus groups varied between five and nine. The focus group sessions lasted between 70 to 90 minutes and were conducted from June to December of 2009. To prevent issues of power differences in each group, the moderator encouraged equal participation in the group and guided the discussion so that every respondents was able to express his or her opinion. A moderator led the focus groups and used a discussion guide with a few open-ended questions (appendix). To gain a deeper understanding of the experience that were most important, relevant, and problematic for the respondents, and according to the principles of the Grounded methodology, we edited the discussion guide based on a continual analysis and comparison of collected data before moving on to the next focus group. Data collection and analysis occurred simultaneously.

### Data analysis

The focus group discussions were recorded and transcribed verbatim. Memos were also noted during the research process or immediately after data collection. In accordance with grounded theory, transcripts and memos were analysed several times and line-by-line (open coding) to find the words or phrases used by the respondents to describe their challenges and how they are trying to solve it. In the final stage of data analysis, we manually sorted the codes into larger categories and sub-categories (selective coding). All codes were compared. This is what is called the "constant comparative method" [17,18]. A core category and four sub-categories of what was most important for the respondents were found. Quotes correlating to each of the categories are collected in separate tables and cross-referenced to focus group numbers and the respondents' professions.

### Ethical considerations

To save time, we asked the administrators of the community health services in question to recruit all relevant HCP's for the focus groups. Use of this procedure obviously leads to concern regarding the issue of coercing respondents to participate. However, a letter was given to all respondents informing them that participation was on a voluntary basis and that they could withdraw from the study at any time without any further obligation. All respondents gave written consent that they would participate. All gathered material has been treated anonymously. More detailed characteristics of the patients and the communities in Table 1 are omitted to ensure the anonymity of the patients, respondents, and the communities. The study was approved by the Norwegian Social Science Data Services (20781) and Regional Committee for Medical Research Ethics (REKNORD 5/2009).

## Results

The core category in our findings describes how the community health care services, at both administrative level and in practical care, experience the challenge of "being between a rock and hard place." Furthermore, we found four sub-categories describing the community health care services' challenges related to the core category. When applicable, the respondents gave proposed solutions to solve problems. We found no variation in the experience of HCP's caring for HMV-dependent children compared with those caring for HMV-dependent adults. Discussions in the focus groups uncovered very detailed, gripping, and emotional stories of the daily struggles to help these patients and their family members to live a good, meaningful, and valuable life:*"You get extreme respect for them. You don't want to go and complain about small things when you see what they have to struggle with. You want to fight together with them. You want to be there until the bitter end." (4/Personal Assistant)*

Many of the respondents expressed that, despite all the challenges they had been subjected to, they had gained unique, exciting, and learning rich experience that they did not regret obtaining. Many felt strong understanding of what a huge challenge it is to care for HMV patients in their own homes. Much was at stake and the smallest mistake could lead to serious and even life-threatening consequences. Some of the respondents mentioned that they could understand the family members' reactions and behavior and how they would have reacted the same way if put in the same situation.

### "Between a rock and a hard place"(core category)

To be "between a rock and a hard place" describes the dilemma of being in a position in which one must choose between two unpleasant alternatives, without the opportunity to satisfy all the implicated parties' needs. In this study, respondents described this dilemma in different ways because of the different roles experienced caring for HMV patients. Home care administrators were mostly concerned with describing the discrepancies between the hospital's, the patients', and the families' expectations for the community to have adequate personnel competence, the necessary resources in place, and the daily struggle to keep it all at a professional level. Because of the high turnover rate of HCP's, the administrators constantly focused on staff recruitment, but most applicants were in fact unlicensed caregivers. The collaboration with the specialised hospitals was described as good, but the community workers did, however, complain about not being sufficiently involved in planning and decision-making. Another dilemma arising was the responsibility the communities had for many other patients in the community, while having to prioritise HMV patients because of life-support treatment. Despite this prioritising, the communities claimed that it did not affect the lives and health of the other patients, but instead the workload became heavier for the HCP's.

The HCP's providing the practical and everyday care in the patient's home described the dilemma of having to answer to several counterparts simultaneously: the hospital responsible for the technical dimension of the treatment, the community as their employer, the families, often deeply involved in the care, and their own opinions about what was best for the patient. The following sub-categories describe these dilemmas in more detail, and in addition, the respondents proposed solutions to some of them.

#### Quotation examples "Between a rock and a hard place"(core category)

• *"What can the community contribute then? And what should the community get involved in? We are standing here between a rock and a hard place. We receive directives from the hospital: "Please take this patient". And the parents are completely stressed out and on the tips of their toes. And it's an incredible difficult start to a collaborative relationship." (4/RN/previous leader)*

• *"The patient and the family did not accept all of the guidelines we had there. We were not allowed to do what we were directed to do. I felt we were very much in between a rock and a hard place." (2/Certified Nursing Assistant)*

• *"The technical issues, that you're not involved in the testing, the medical issues, what happens in the hospital. We're told to try this out for a period. This can be anything from antibiotics to new pressures and changes to the ventilator. And to initiate a single nursing procedure, you have to discuss with the parents and they try to challenge you. In a way, you're never able autonomously to make decisions. You hear the same from others all the time. This is why we feel we are stuck between a rock and a hard place." (5/RN)*

### To be a guest in the patient's home

All of the respondents were most interested in describing the challenges associated with working in the HMV patient's private home. Working inside a private residence was described as the biggest challenge and was the main trigger for the other challenges they described. The HCP's experienced they were merely guests in the patients' homes, and this was something they had to be constantly aware of while at work. They held a low profile in order not to disturb peace in the home and thereby to avoid conflicts. The staff consciously kept a neutral demeanor, did not stand out, stayed quiet, closed their ears, and held their tongues. They were, after all, in the patient's home and had to behave in keeping. The relationship with the patient's family was always the most difficult; less so was the patient himself/herself or the technical aspects of the care. Working alone with an HMV patient in a private home was described by the HCP's as both socially and professionally lonely. In many instances, the patient's level of communication was limited, contributing to the HCP's loneliness. Loneliness also refers to how the HCP's were only able to discuss clinical questions with other colleagues during shift changes. Boredom could be explained by the mundane everyday tasks, or because some patients were unwilling to participate in outdoor activities. To solve the issues relating to the patient living in his or her own home, respondents from each community recommended that the patients be placed in assisted-living facilities to improve HCP's work environment.

#### Quotation examples "to be a guest in the patient's home"

• *"The biggest challenge for us is to be in a private home. This has to do with meeting the family in their residence and complying with them. This is the biggest challenge of this job." (5/Social Educator)*

• *"I steadily learned that I was crossing a threshold into another home. I think this is important to think about. This is where they actually live. I am, in a way, a guest here. And I knew my role as a guest. Being humble with regard to what their wishes were, as best I could." (3/Critical Care RN)*

• *"You're unable to get people to work with this. No one wants to work there. The patient on a ventilator is very demanding in how he wants the assistance. And the spouse. The demands are too big and too negative, so no one wants to work there." (3/RN)*

• *"We thought they could live collectively so they could share personnel and a similar environment. Where they are able to combine their private lives in their personal living space, that is their own private arena, but where the personnel has a working environment with other patients and other personnel, so that they can be taken care of. I don't think we will be able to manage more HMV patients living completely isolated in the house they lived in when they were well." (3/RN/Cummunal Director)*

### To be accepted or not?

In addition to attending formal courses, in collaboration with the community health services and the specialised hospitals, some of the HCP's had to undergo a sort of informal approval from expert family members - which many deemed problematic. The respondents sensed that the intention of this informal approval was a way for the family members to find out if the HCP's had the necessary skills, and further if they were suitable for the job. The HCP's believed they were put through a test, despite the community health service's leadership acknowledging they were qualified to fulfill the job. Others described that they were well accepted and regarded as an extension of the family, thereby putting the HCP's in between two roles: professional and friend. This was a difficult position to be in.

#### Quotation examples "to be accepted or not"

• *"It makes things worse when we don't have enough people who can visit him and be accepted there. You're first accepted when you've received training. There is always an incredibly high threshold to come in and receive training. It is always a struggle. And we can't continue like this, having too little people when the need for people is so huge." (1/RN)*

• *"I don't think very many people from the team have quit because of the patient, or because the patient saw them as useless. The family itself has labeled them as useless." (2/RN)*

• *"You're caught in between the two roles of being a professional and being a friend. In every role, you're caught somewhere in between. You have no clear role in relation to anything. In everything we do. We don't actually decide anything. And in this way you're caught between the patient and the parents." (5/Social Educator)*

### Who decides?

The HCP's working directly with the patient expressed enormous frustration with regard to who decided on the medical treatment. Was this the specialised hospital, the patient, the family, the community health administration, or the HCP in question? The HCP's believed they possessed the competence to make the professional decisions but had to eventually consult with the family, and many times compromise on their decisions with persistent family members who had become experts in care. The disagreements and obscurity surrounding these decisions especially affected the unlicensed carers; therefore, the respondents suggested that the boundaries for what the families should be involved in needed to be clarified. Many of the respondents thought it was very unfortunate that some family members were employed as part of the health care team, leading to the double role of both caring family member and care provider. This was often the source of conflicts, eventually ending up in the resignation of a HCP; therefore, most of the respondents proposed that family members schould not be employed as part of the health care team.

#### Quotation examples "who decides?"

• *"She (the wife) claimed rights she did not have. This was because it was in her home, so she had a completely different role than the other employees coming from the outside. And because of this, she became a type of informal leader and did the decision-making." (3/RN/Communal Director)*

• *"Who decides? God only knows. I have been fighting with them for years. In the end, it was always them." (4/RN/previous leader)*

• *"What I thought was very unfortunate was to employ family members as part of the health care team, because this became a very difficult role. She went and checked anything she was unsure about. And it was very difficult to be controlled in work-related situations. And this quickly turned into conflicts." (3/Critical Care RN)*

### How much can we take?

The majority of HCP's experienced conflicts with families while working in the patient's home. Some felt harassed, but believed there was a limit to how much one could get involved. In these situations, the HCP could not distance themselves because the patient's condition demanded them to be constantly present. All of the respondents knew the reasons the previous HCP had resigned. This was not because they did not enjoy working with the patient, rather the mental burden of being alone in these situations, without the possibility to reach out for support from fellow co-workers.

#### Quotation examples: "how much can we take?"

• *"Where do we draw the line? I tell the family member that I do not accept the way you behave when you are here together with your spouse. So, you were caught in the middle...the family member had his or her spouse in the home, while I had to care for my employees." (1/RN/nurse manager)*

• *"Those at work deserve a good work environment as well. Finding the threshold of where the limit goes. But I have been blamed for crossing this line many times." (2/licenced vocational nurse)*

• *"If only the family had a little different attitude, another way to cooperate. I've thought about this many times. If the relationship with the family were different, then I think those eight years with the patient could have been pure bliss." (2/RN/Communal director)*

## Discussion

This study has illustrated that caring for HMV patients with complex needs represents a wide range of immense challenges for community health services. The respondents in this study described the dilemma of being a guest in the patient's home and having to behave accordingly. Being in this position can create obstacles when providing care, as confirmed by previous studies [6]. Similar to previous research [8], our study also highlights how considerable family member involvement as informal carers and experts challenges the role and authority of HCP's. Understandably further frustration is experienced when family members are employed as part of the care team for loved ones. This type of system is apparently exclusive to Norway, as we did not find similar descriptions in the literature review. In this study, these relationships had a distinctly negative influence on the HCP's professional and psycho-social work environment when working in the patient's home. As a consequence of this, the study revealed that many of the HCP's could not continue working in the patient's home and eventually quitted. This same phenomenon is also described in another study [9]. HCP recruitment was difficult, either because potential job seekers did not picture themselves with such a unique type of care or because they had heard how difficult it is to work in the patients' homes. Whereas it is important that HCP's have competence tailor-made for this special patient group [1,19], the communities we studied often recruited unlicensed carers. The high turnover rate and lack of competent and experienced HCP's led to constant focus on recruitment and training of new employees. These challenges stand in contrast to what HMV patients describe as imperative to live a good and active life: competent HCP's and continuity in care [11]. In our study, the respondents cited how the lack of continuity and competence among HCP's also had a negative impact on the family members. It increased the burden on family members because they had to step in and do much of the work themselves or they could not trust the caregiver, especially when the family was not present. These issues have been documented in other studies [20-22].

What can be done to reduce the consequences related to the challenges revealed in this study and what proposed solutions do the respondents in this study suggest? Similar to the findings from previous research [8], the results from this study emphasise the need for dialogue regarding the boundaries of family member involvement in this type of technically advanced care. If the solution includes limiting the involvement of family carers, something we sensed that the respondents in this study wanted, this will probably prove difficult for the families to accept. Family carers are aware that their competence level may well exceed that of the other carers and that their expertise is important for the well-being and survival of the ventilator-dependent patient [4]. Additionally, finding an alternative to family carers could be difficult because professional support for this type of specialised care is not always readily available [20], which was also the case in the communities we studied. Instead, the HCP's should recognise the family members' competence and be willing to learn from them [4].

Initiatives to improve the HCP's professional and psychosocial work environment seem to be important to ensure continuity and competence. Several studies have pointed out the importance of supporting the family carers' emotional and psychosocial needs [12,23-25]. Our study identifies this same need in the HCP's and supporting the families' emotional and psychosocial needs should be done systematically, which was not being done in the communities we studied.

The respondents in our study recommended that this complex group of HMV patients should be treated together in an assisted-living facility to avoid the challenges associated with caregivers working alone in the patients' homes. This recommendation corresponds with a recent statement from the Norwegian Director of Health, which implies that communities should not have the responsibility or obligation to care for these types of patients in their homes because of the enormous challenges associated with its provision [26]. The respondents argued that this would improve the work environment by strengthening both the social and professional work cultures. By working with several patients an increased variation in work would be achieved, which could prevent employee burnout and reduce turnover rates. Reduced employee turnover rates could lead to increased competence, something that would most certainly benefit the patient. The respondents defended the use of assisted-living facilities by claiming that the community health care services' role in decision-making and setting limitations and guidelines would be strengthened, which would reduce costs. Another benefit was the possibility for family members to live a more normal life by relieving them of some of the workload associated with caring for an HMV-dependent family member. Despite these benefits, the respondents believed both the patients and the families would strongly oppose to the idea of not having the right to live in their own homes. Patients and parents of HMV-dependent children want to live at home [11,20,27]. It gives the HMV patient a feeling of freedom and security [28], even though it can be stressful for patients and families to have an unfamiliar health care worker in their homes [1,11,29].

Fulltime HMV care can be considerably expensive [30] and disagreements and obscurities of the financial responsibilities are described [13,31]. Because of large regional differences in the provision of HMV [32], some communities carry a much heavier financial burden than others. In the present study, expenses were never mentioned as a problem or as a source of the challenges the municipalities described. This is probably because health care financing varies from country to country and because the Norwegian health care system, including both specialised hospitals and community health care services, is publicly financed. In addition to public financing, the Norwegian government has developed special funding for community health care services caring for complex HMV-dependent patients. Despite the availability of financial support, the Norwegian Director of Health has recently signalised a possible reduction in the provision of fulltime-monitored HMV because of the high demand for HCP resources thereby generated. No literature was found to support that this is a prioritisation other countries are also considering.

### Possible limitations

The sample in this study was small, as in most qualitative studies. Despite this limitation we believe this study draws an accurate picture of the current situation in Norway. We believe the results of this study may have transferability to other types of advanced in-home treatment, such as tracheostomies, dialysis, parental nutrition, and intravenous medications. One must take into consideration that the results of this study may not be transferable to other countries because of the differences in treatment, delegation of responsibilities, organising, and financing of HMV. Neither the patients nor the families have validated the results of this study because we focused only on the experience of HCP's. However, we recently conducted a study involving all the five communities used in the present study to investigate the experience of family members of HMV patients with complex needs (manuscript submitted). Interestingly, we found that there is a large gap between family members' expectations and what the community health care services are able to provide, even when almost unlimited resources are available.

## Conclusions

This study illustrates how home care for ventilator-dependent patients with complex needs presents a wide range of immense challenges for community health care services, both at administrative level and for the health care personnel in direct contact with the patient and his or her family. These challenges can have a negative impact on community HCPs' work environment and the community health care services' provision of professional care. The results of this study point towards the need to define the roles of family caregivers and HCP's, and also to find solutions to improve their collaboration. A debate considering whether family members should be formally employed as carers for loved ones is also needed. The work environment of HCP's in patient's homes must be improved to ensure the necessary competence and quality of care. Specialised hospitals should allow community health care services to be more involved in both the adaptation of HMV and long-term follow-up care. The study also shows the need for more dialogue concerning eligibility requirements, rights, and the limitations for patients in the provision and use of HMV in private homes. Further research exploring the experience of HCP's in other countries caring for individuals dependent on continual care and highly advanced technology is needed.

## Appendix

### Interview guide used for the first focus groups

1. What kind of problems and challenges do the community health care services encounter when caring for individuals dependent on home mechanical ventilation and highly specialised care?

2. Who decides on the treatment at home?

3. Describe the cooperation with the family members?

4. What is the positive side of working with these patients?

5. Do you have any suggestions for how these patients might be cared for in the future?

6. Is there anything else you would like to add about community-based care for HMV-patients that we have not discussed here today?

## Competing interests

The authors declare that they have no competing interests.

## Authors' contributions

KD, BSB and EWN were responsible for the study conception and design. KD and BSB performed the data collection. KD performed the data analysis and drafted the manuscript. BSB and EWN made critical revisions to the paper for important intellectual content and supervised the study. All authors read and approved the final manuscript.

## Pre-publication history

The pre-publication history for this paper can be accessed here:

http://www.biomedcentral.com/1472-6963/11/115/prepub
